# Neurally adjusted ventilatory assisted ventilation compared to pressure support during post-operative weaning of hepatic patients undergoing major abdominal surgeries: a randomized control trial

**DOI:** 10.1186/s12871-025-03159-y

**Published:** 2025-06-26

**Authors:** Eman Sayed Ibrahim, Hatem A. Attalah, Essam A. Eid, Rasha F. Elshoney, Amany A. Sultan

**Affiliations:** 1 Department of Anaesthesiology, Intensive Care and Pain Management, National Liver Institute – Menoufia University, Shebeen El-Kom, Egypt; 2 Department of Anaesthesiology, Intensive Care and Pain Management, Faculty of Medicine, Menoufia University, Shebeen El-Kom, Egypt

**Keywords:** Pressure support ventilation, Neurally adjusted ventilatory assist, Patient-ventilator asynchrony

## Abstract

**Objective:**

Patient-ventilator asynchrony (PVA) is a discrepancy between the patient’s demands and the ventilator. Pressure support ventilation (PSV) is the most popular mode of partial ventilation. Neurally adjusted ventilatory assist (NAVA) tailors the level of assistance to the diaphragm’s electromyographic activity. We aimed to evaluate the PVA during NAVA versus PSV in hepatic patients undergoing Whipple operation.

**Methods:**

This study was a prospective, randomized, controlled double-blind trial. 120 adult Child-Pugh A and B hepatic patients of both sexes aged 18 to 65 years, undergoing Whipple operation, completed the study. We excluded severely debilitated patients and contraindications to the placement of the NAVA probe. 60 patients in the PSV (group) received PSV weaning mode, and 60 in the NAVA (group) received NAVA weaning mode. Outcome measures were asynchrony index, duration of mechanical ventilation MV, application and duration of post-extubation non-invasive CPAP, total sedating drug doses used, and ICU stay.

**Results:**

The total asynchrony index was lower in the NAVA group than the PSV group, 6.12 ± 1.51 and 7.06 ± 1.25%, respectively, *P* < 0.001(95%CI 0.43 to 1.44). Cases with total ASI ≥ 10% were lower in the NAVA group than the PSV group, 6 and 16, respectively, *P* 0.02.The NAVA group had better oxygenation than PSV group, PaO_2_ and PaO_2_/FiO_2_ ratio_s_ were 208.53 ± 14.61 vs. 188.8 ± 25.55 mmHg, *p* < 0.001(95%CI -27.25 to -12.20) and 521.32 ± 36.52 vs. 472.01 ± 63.88, *p* < 0.001 (95%CI -68.12 to -30.50) respectively. The NAVA group had a lower duration of MV (4.05 ± 0.29 vs. 4.62 ± 0.52 h; *P* < 0.001(95%CI 0.41 to 0.72), auto-PEEP occurrence, and total doses of fentanyl and propofol than the PSV group.

**Conclusions:**

NAVA serves better patient-ventilator interaction and synchronization than PSV, as indicated by a lower total asynchrony index, a reduced number of patients with total ASI ≥ 10%, the total doses of sedation used, the duration of MV needed, minimized need for post-extubation CPAP, and better oxygenation.

**Trial registration:**

Menoufia University Faculty of Medicine research ethics committee IRB (N. and date 3/2020/ANET9), and National Liver Institute Menoufia University Institutional Review Board (NLI IRB 000140140). The study was retrospectively registered at the Pan African Clinical Trial with identification number PACTR202401894086611 on 26 January 2024.

**Clinical registration:**

The study was registered at the Pan African Clinical Trial with identification number PACTR202401894086611.

**Supplementary Information:**

The online version contains supplementary material available at 10.1186/s12871-025-03159-y.

## Introduction

Post-operative care for hepatic patients undergoing long-duration major abdominal surgeries includes mechanical ventilation in the intensive care unit (ICU), particularly in the immediate post-operative period, with every attempt to reduce the period of ventilation. Hepatic patients, particularly those with acute-on-chronic liver failure or acute liver injury, have physiological changes that increase hazards of mechanical ventilation, such as increased intra-abdominal pressure and diaphragmatic dysfunction attributable to ascites and hepatosplenomegaly that reduces the diaphragm and lung compliance, resulting in atelectasis that makes the lungs more prone to ventilator-induced lung injury [1]. The pressure support (PSV) is the most popular mode of partial ventilation, in which a steady, pre-set pressure level assists each inspiration independently of the patient’s inspiratory effort [2, 3]. The discrepancy between the level of assistance and the patient’s respiratory drive can consequently occur, resulting in: respiratory discomfort, lung overdistension, and patient-ventilator asynchrony [4, 5]. Neurally adjusted ventilatory assist (NAVA) is a mode of mechanical ventilation that tailors the level of assistance delivered by the ventilator to the electromyographic activity of the diaphragm. An EDI catheter equipped with an electrode array captures the electrical discharge of the diaphragm when positioned in the esophagus [6, 7]. Patient-ventilator asynchrony (PVA) is a discrepancy between the ventilator and the patient’s respiratory system demand. PVA is notably harmful in hepatic patients as it can lead to altered distribution of ventilation, exacerbating existing V/Q mismatches and raising the work of breathing. Over-assistance can lead to ventilator-induced lung Injury [8]. 

Definitions for asynchronies were set by Thille et al. [9]. Ineffective triggering (untriggered breath) is a concurrent decrease in airway pressure and an increase in airflow without assisted cycle. Auto-triggering is a ventilatory breath not preceded by a lowering in airway pressure. The Double triggering, two cycles are disconnected by an expiratory time less than half the mean inspiratory time. A short cycle is an inspiratory time of less than half the mean inspiratory time. The prolonged cycle is an inspiratory time greater than double the mean inspiratory time. The total asynchrony index (ASI) is the total of asynchronies (double-triggered breaths, untriggered breaths, and prolonged cycled breaths) divided by the total number of breaths (the sum of triggered and ineffectively triggered breaths) [9]. 

This study aimed to compare neurally adjusted ventilatory assisted ventilation (NAVA) to pressure support ventilation (PSV) effects on the asynchrony index during weaning of hepatic patients undergoing major abdominal surgeries. Secondly, the number of cases with total ASI ≥ 10%, the need and duration of post-extubation non-invasive mechanical ventilation, doses of sedating drugs used, oxygenation, and ICU stays were recorded.

## Materials and methods

### Approval and trial registry

After approval of the local Menoufia University Faculty of Medicine research ethics committee IRB (N. and date 3/2020/ANET9), and National Liver Institute – Menoufia University Institutional Review Board (NLI IRB 000140140). The study was retrospectively registered at the Pan African Clinical Trial with identification number PACTR202401894086611 on 26 January 2024. The study was conducted in the Anesthesiology department, National Liver Institute, between May 2022 and September 2023.

### Study design and patient characteristics

This double-blinded randomized controlled trial (RCT) was conducted on 120 hepatic patients who were equally randomized into two groups, group 1 (PSV) received post-operative PSV weaning mode, and group 2 (NAVA) received post-operative NAVA weaning mode. All experiments were performed following relevant guidelines and regimes. Informed consent and ethics approval to participate were obtained from patients.

The study incorporated adults above 18 years old. Hepatic patients (Child-Pugh A and B) undergoing Whipple operation require post-operative mechanical ventilation. We excluded patients, Child-Pugh C hepatic patients, severely debilitated patients, and contraindications to placement of the NAVA esophageal tube (i.e., rupture of esophageal varices or esophageal malformation). Our study adhered to and complied with the Helsinki Declaration (https://www.wma.net/policies-post/wma-declaration-of-helsinki/*).*

Preoperative surveillance included hematological screening (hematocrit level, serum electrolytes, and blood grouping), biochemical liver and renal tests, prothrombin time, and international normalized ratio. In addition, electrocardiography and chest X-ray were ordered if indicated. Premedication, intraoperative general anesthesia induction, and maintenance were the same for all patients.

### Randomization and blindness

Patients were randomly allocated into one of the two study groups: group 1 (PSV) received post-operative PSV weaning mode, and group 2 (NAVA) received post-operative NAVA weaning mode. The randomization with a 1:1 allocation ratio to ensure equal distribution of participants between the studied groups was performed using a computer-generated randomization sequence from an online program (http://www.randomizer.org). Concealment for patient allocation numbers was performed using opaque, sealed envelopes. Each envelope involved the group recruitment corresponding to the randomization number, and the envelope was only opened promptly after the completion of the surgery at ICU admission. Blindness was maintained for the patients and the person who conducted the statistics.

### Sedation strategies

The patients were sedated according to hemodynamics, patient condition and demand, and departmental policy with variable rates of infusion of fentanyl infusion (1–2 mcg/kg/min) and propofol infusion (25–75 mcg/kg/min) to maintain moderate sedation as revealed by Richmond Agitation and Sedation Scale (-3) [10].

### Ventilation strategies for weaning

After completion of the surgery, patients were discharged to the ICU, intubated and sedated with controlled mechanical ventilation till stabilization of the patient. Muscle relaxant reversal by neostigmine, depending on electromyography-based quantitative monitoring to confirm the train-of-four (TOF) ratio ≥ 0.9. Then, the partial ventilator support mode of weaning according to each group, either PSV or NAVA, was applied.

NAVA and pressure support levels were set according to the defined group to achieve a tidal volume of 6–8 mL/kg of ideal body weight.

In both groups, NAVA and PSV were maintained except that the patient had criteria for shifting to controlled mechanical ventilation or weaning and subsequent extubation.

### NAVA and PSV workflow and settings

First, make the Edi module function check and select the right Edi catheter. We used an EDI catheter 16 Fr 125 Cm.

Measure the NEX [distance from the bridge of the nose to the earlobe and the xiphoid process], and calculate the insertion distance (Y) according to the formula:

Y NEX in Cm x 0.9 + 18 for nasal insertion and Y NEX in Cm x 0.8 + 18 for oral insertion. Put the EDI catheter in water for a few seconds. Connect the EDI catheter to the EDI cable. Verify the position using the ECG waveforms. Verify that QRS and P waves are present in the uppermost leads, P waves are hidden, and the QRS amplitude is decreased in the lower leads. Mark and secure the EDI catheter at its final position.

While using NAVA, the assist pressure delivered (in CmH2O) is modified by multiplying the delta EDI (electrical activity of the diaphragm) (Edi peak – Edi min) (expressed in µV) by a relative coefficient, defined as the NAVA level (expressed in CmH2O/µV). NAVA level renders a type of (gain factor): how much CmH2O the patient will receive per µV Edi. The NAVA peak (Ppeak) inspiratory pressure is calculated following this equation:

$$\eqalign{{\rm{NAVA}}\,{\rm{P}}\,{\rm{peak}} & {\rm{ = the}}\,{\rm{assist}}\,{\rm{pressure + PEEP}} \cr & {\rm{ = NAVA level x }}\left( {{\rm{Edi peak -- Edi min}}} \right){\rm{ + PEEP.}} \cr} $$

The initial NAVA peak pressure (Ppeak) is estimated before starting ventilation with NAVA mode in the NAVA preview window in the SERVO-i ventilator, known as estimated peak pressure (PEst.). Adjust the NAVA level (increase or decrease) till the peak pressure curve in the selected conventional mode before ventilating the patient with NAVA becomes congruent to the estimated peak pressure (PEst.) curve, then start ventilation with NAVA.

The NAVA Edi trigger detects an increase in Edi and should be set to a level higher than the variable background noise, where it is commonly less than 0.5 µV, so the default setting is 0.5 µV (0–2 µV) [11–13]. 

NAVA mode parameters: NAVA level (Cm H2O/µV): Mentioned above. PEEP (Cm H2O): The optimal PEEP for each patient is to achieve the highest static compliance. The fraction of oxygen concentration (FiO2%): 40% and trigger Edi (µV): Mentioned above.

**Backup ventilation: Mode**: pressure-controlled ventilation.

**Pressure support settings in PSV group**: PS above PEEP (Cm H2O): 5–20 Cm H2O to obtain a TV of 6–8 ml/kg of ideal body weight. Trigger sensitivity: (− 1 cm H2O), PEEP (Cm H2O): the optimal PEEP for each patient and oxygen concentration (FiO2%): 40%.

### Criteria for shifting to assist-control MV in the two studied groups

Respiratory distress, hypoxemia, or hypercapnic acidosis, even though refinement of ventilator settings, marked decrease in the blood pressure, shock or arrhythmia, agitation, or patient-ventilator asynchrony. Once the parameters for shifting to assist-control mechanical ventilation were regained, partial ventilatory support with NAVA and PSV was reinstituted according to the patient group.

### Weaning protocol

Decrease the level of assistance gradually in both groups by decreasing the pressure support level in PSV group to 5 CmH2O and the NAVA level in NAVA group 0.5 and if weaning criteria fulfilled: (1) SpO2 > 92% with FiO2 ≤ 50%, (2) PEEP less than 5 cm H2O, (3) No infusion of vasopressor agents, (4) Haemodynamic stability and (5) No need for further surgical interference. We stopped sedatives until there were adequate responses to simple commands, a spontaneous breathing trial was performed (Breathing spontaneously for 30 min to 1 h), then disconnected from the ventilator on a T piece, or in pressure-support ventilation with an inspiratory pressure of 7 cm H2O and PEEP 5 Cm H2O. The test was interrupted if signs of poor tolerance occurred: respiratory rate > 35/min, SpO2 < 90%, or arterial systolic blood pressure above 180 or below 90 mmHg. If no evidence of improper tolerance, the patient is extubated.

### Data collection and outcome measures

Primary outcome: Total asynchrony index.

Secondary outcomes: The index of each type of asynchrony (ineffective triggering, auto-triggering, double triggering, short cycle, and prolonged cycle). The number of cases with severe asynchrony (total ASI ≥ 10%). Lung mechanics: driving pressure DP, PEEP, peak inspiratory pressure PIP, mean airway pressure Pmean, compliance, hemodynamics, and use of vasopressors (number of patients and total dose). Also, total sedation requirements (types and total dose required), duration of mechanical ventilation, application and duration of post-extubation non-invasive mechanical ventilation, ICU, and hospital length of stay were recorded.

These data were collected every hour after the application of either PSV or NAVA till the extubation of the patient. T1 is the first hour, T2 is the second hour, T5 is the fifth hour, etc., and average reading is the average of all measured values for each parameter in all recorded hours. We calculated the asynchronies for five minutes per hour of ventilation until weaning from the ventilator. We recorded the number of each type of asynchrony found during these five-minute periods and calculated the number of triggered breaths (the respiratory rate [breath per minute] × 5). As asynchronies are not static phenomena, their occurrence and severity can vary significantly every time, so repeated monitoring is important to get an overall picture.

$$\eqalign{{\rm{The}}\, & {\rm{index}}\,{\rm{of}}\, {\rm{each}}\,{\rm{type}}\,{\rm{of}}\,{\rm{asynchrony }} \cr & {\rm{ = }}{\matrix{{\rm{Total}}\>{\rm{number}}\>{\rm{of}}\>{\rm{the}}\> \hfill \cr {\rm{selected}}\>{\rm{asynchrony}} \hfill \cr} \over \matrix{/{\rm{Total}}\>{\rm{number}}\>{\rm{of}}\>{\rm{cycles}}\> \hfill \cr ({\rm{no}}.\>{\rm{of}}\>{\rm{triggered}}\>{\rm{and}}\>{\rm{ineffective}} \hfill \cr {\rm{triggered}}\>{\rm{breaths}}).\> \hfill \cr} } \times 100 \cr} $$

$$\eqalign{{\rm{The}}\,{\rm{total}}\, & {\rm{asynchrony}}\,{\rm{index}}\,\left( {{\rm{ASI}}} \right)\,{\rm{\% }} \cr & {\rm{ = }}{{{\rm{Total}}\>{\rm{number}}\>{\rm{of}}\>{\rm{asynchronies}}} \over \matrix{\>{\rm{Total}}\>{\rm{number}}\>{\rm{of}}\>{\rm{cycles}} \hfill \cr ({\rm{no}}.\>{\rm{of}}\>{\rm{triggered}}\>{\rm{and}}\> \hfill \cr {\rm{ineffective}}\>{\rm{triggered}}\>{\rm{breaths}}) \hfill \cr} } \times 100 \cr} $$

#### Determining driving pressure

We performed an acceptable inspiratory hold technique using predetermined criteria: duration of the occlusion > 2 s, airflow equal to 0 mL/s (flat tracing on the airway pressure curve), and absence of visible thoracic or abdominal movement. If patients had an acceptable breath-hold maneuver, we recorded the plateau pressure. We determined driving pressure as the difference between the plateau and positive end-expiratory pressure.

### Validity of the method we utilized to reveal asynchrony

Values of the total asynchrony index and all other asynchronies were detected using flow and airway pressure signals only by 2 independent observers, and simultaneously, they were detected using flow and airway pressure signals together with EDI signals by 2 independent observers. Based on 15 recordings of the asynchronies by both techniques and on analyzing the collected data, there was a no significant difference among the two observers’ readings using flow and airway pressure signals only (*P* 0.62) and the two observers readings using flow and airway pressure signals together with Edi signals (*P* 0.71). There was a highly significant positive Pearson correlation between the readings of the asynchronies measured by both techniques in both observers (*P* < 0.01). This result backs the reliability of non-invasive asynchrony revealing based on flow with airway pressure and EDI signals.

### Sample size calculation

Based on Diniz-Silva et al., 2020 [14], who found that the asynchrony index was 0.78% (SD = 1.02) in PSV and 0% (SD = 0.89) in the NAVA group. At a power of 95%, alpha error 0.5 and based on the following formula: *N* = 2(Zα + Z [1-B]) ^2^× SD^2^/d^2^.

N sample size, α = corresponding constant for alpha error (1.96), Z [1-B] = corresponding constant for power of the study (1.63), SD = average of standard deviation, and d = mean difference. The calculated sample size was 58 patients in each group.

### Statistical analysis

Data were analyzed using SPSS (Statistical Package for the Social Sciences software) version 17.0 (SPSS Inc., Chicago, IL, USA). Descriptive statistics were expressed as numbers and percentages (No & %) for qualitative data, and mean (x) & standard deviation (SD) for quantitative data. Analytic statistics: The chi-squared test (χ2) was used to study the association between qualitative variables, and Fisher’s exact test and Z test were used in the analysis of 2 × 2 contingency tables., Student t-test (t) and Mann-Whitney U test used for quantitative variables, Pearson correlation and Spearman correlation (r): to measure the correlation between variables, P-value < 0.05 was considered statistically significant.

## Results

### Comparison of patient characteristics data

**Fig. 1:**
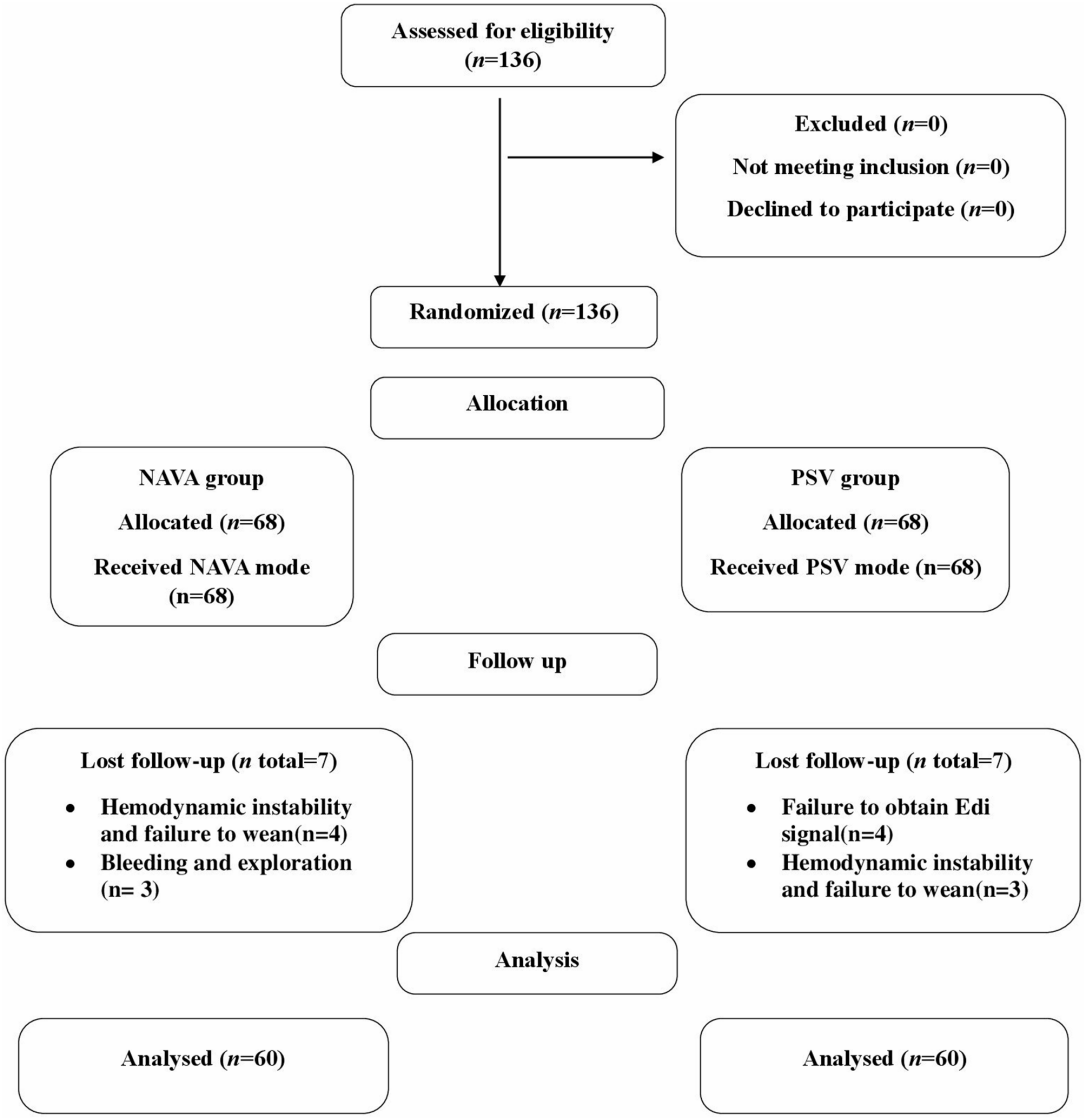
CONSORT flow chart. NAVA: Neurally Adjusted Ventilatory Assisted, PSV: Pressure Support Ventilation

One hundred twenty patients completed the study: sixty patients in the NAVA group and 60 in the PSV group. Fourteen patients dropped out (Fig. 1). Our results showed no significant difference between the PSV and NAVA groups regarding age, sex, height, and ideal body weight (*P* 0.13, 0.12, 0.75, and 0.74), respectively. Our study enrolled 56 (93.3%) vs. 49 (81.7%) patients with Child Pugh class A and 4 (6.7%) vs. 11 (18.3%) patients with Child Pugh class B in PSV and NAVA groups, respectively, with *P* 0.053. The presence and severity of ascites were assessed by ultrasound and abdominal CT scan and in our study only minimal ascites were found in 1 (1.7%) vs. 2 (3.3%) patients in PSV and NAVA groups respectively with *P* = one which is not clinically significant, this minimal ascites could not impact on respiratory mechanics and outcomes of our study. All patients of both groups underwent Whipple operation (Table 1).

**Table 1:** Patients sociodemographic and operative data. Values are mean ± sd or number

Variable	Group 1 PSV (*n* = 60)	Group 2 NAVA(*n* = 60)	Test	*P* value
Age (years)	55.70 ± 8.94	52.92 ± 11.11	t-test 1.51	0.13
Sex (M/F)	36 /24	44 /16	χ2 2.40	0.12
Height (cm)	165.65 ± 4.31	135.38 ± 4.64	t-test 0.33	0.75
Body weight (Kg)	60.35 ± 5.39	60.67 ± 5.06	t-test 0.33	0.74
Comorbidities Negative DM HTN COPD Asthma	30 (50.0) 16 (26.7) 8 (13.3) 8 (13.3) 1 (1.7)	36 (60.0) 14 (23.3) 5 (8.3) 3 (5.0) 2 (3.3)	Z test 0.92 0.21 0.59 1.27 0.0	0.36 0.83 0.56 0.21 1.0
Child Pough Child A Child B	56 (93.3) 4 (6.7)	49 (81.7) 11 (18.3)	χ2 3.73	0.053
Surgery Whipple	60 (100)	60 (100)		
Operative time (hours)	9.71 ± 1.30	9.43 ± 1.56	t-test 1.07	0.29
Total amount of Ringer acetate (liters)	4.78 ± 0.25	4.87 ± 0.39	t-test 1.55	0.13
Total amount of Hydroxyethyl starch (liters)	0.78 ± 0.25	0.75 ± 0.25	U 0.73	0.47
Total amount of packed RBCs(ml)	*N* = 33 618.33 ± 276.16	*N* = 25 647.50 ± 279.59	U 0.58	0.56

NAVA: Neurally Adjusted Ventilatory Assisted, PSV: Pressure Support Ventilation

Data normally distributed; tested by T-test. Data not normally distributed tested by Mann-Whitney U test. Chi-squared test (χ2), Fisher’s exact (Z)

Data was collected every hour, and then the average of the measured values was analyzed.

### Comparison of the asynchrony index in both groups

**Fig. 2:**
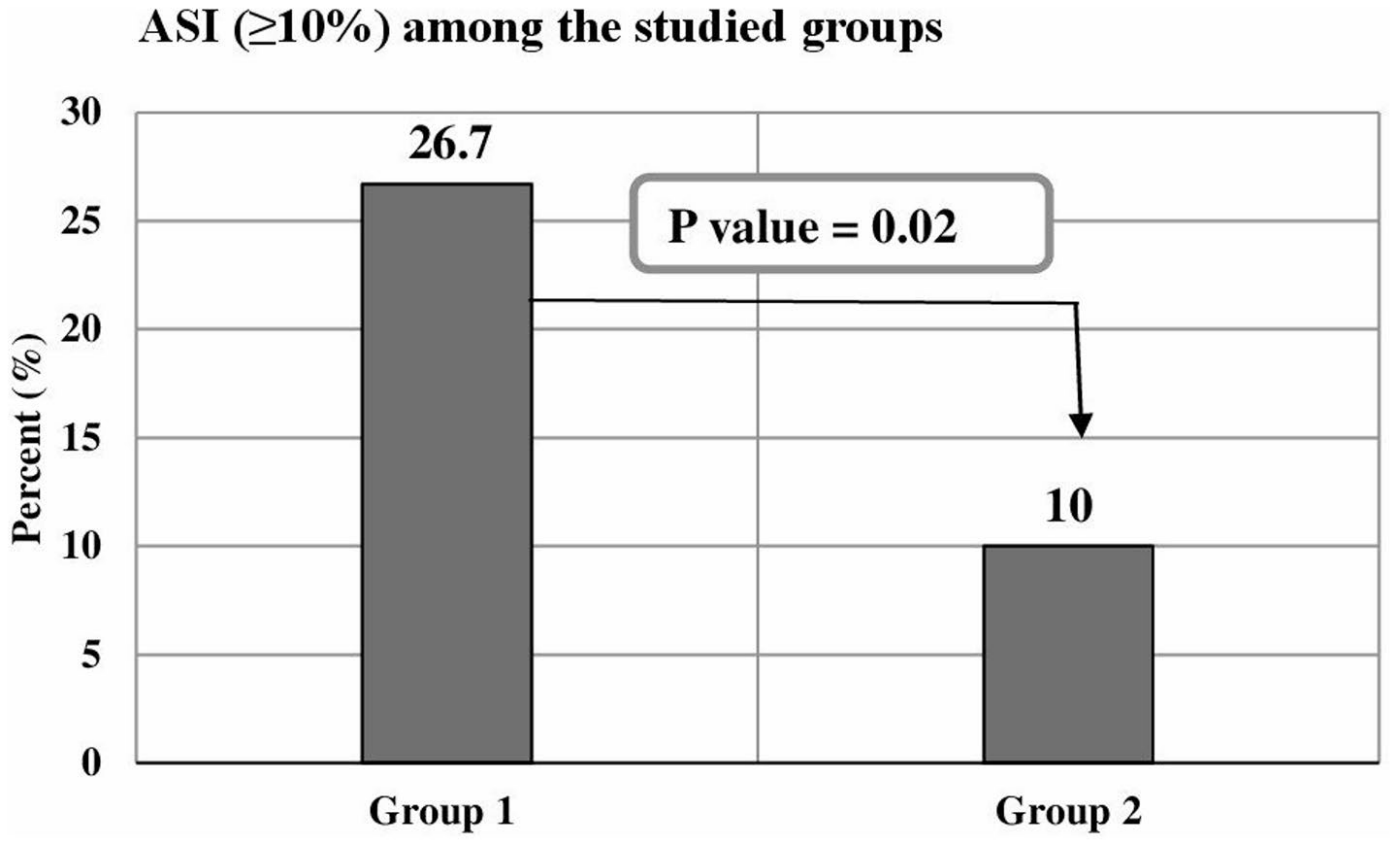
The number of cases with severe asynchrony (total ASI ≥ 10%) in group 1 PSV group (Pressure Support Ventilation) and group 2 NAVA group (Neurally Adjusted Ventilatory Assisted)

**Fig. 3:**
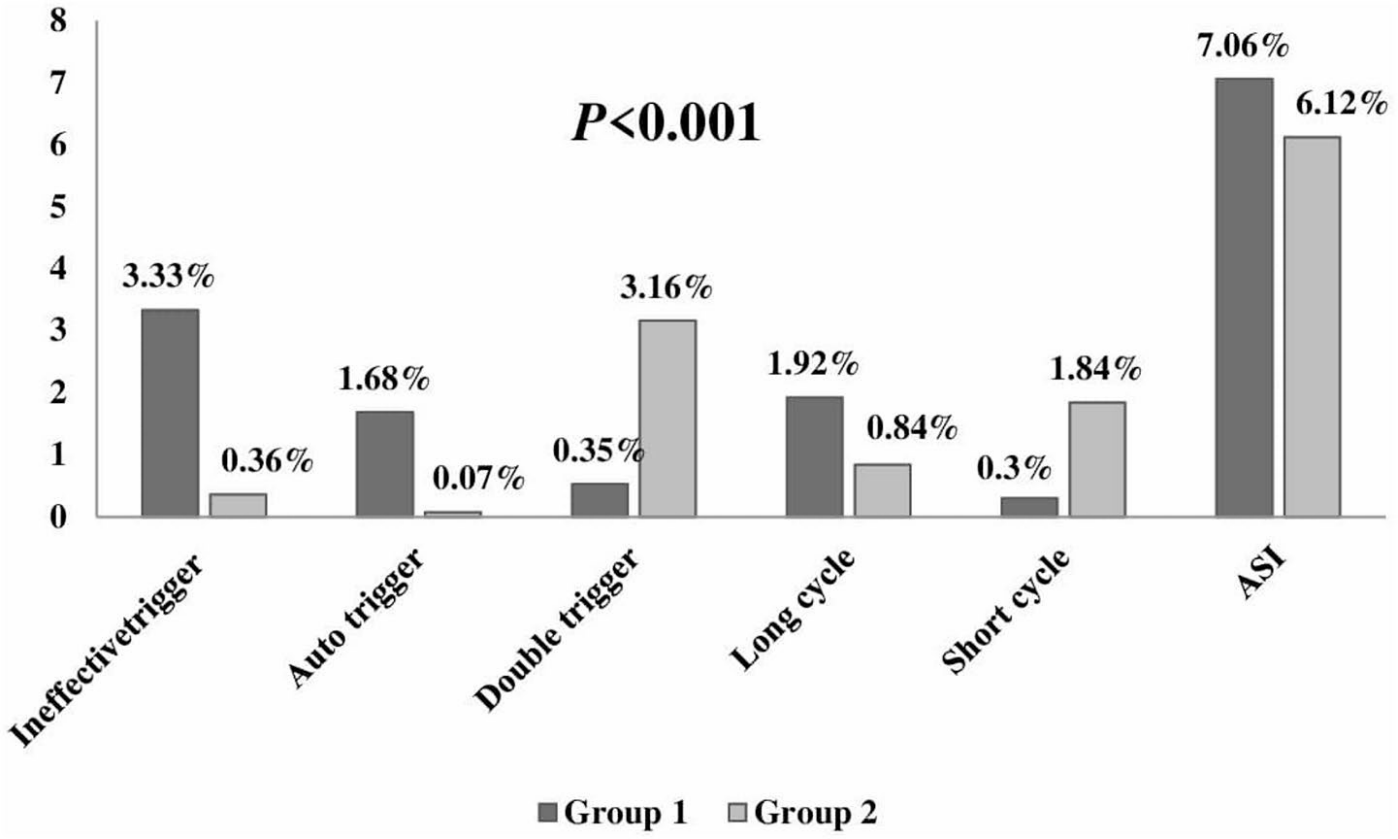
The total asynchrony index, ineffective trigger index, auto trigger index, long cycle index, double trigger index and short cycle index in group 1 PSV group and group 2 NAVA group

The number of cases with severe asynchrony (total ASI ≥ 10%) was statistically greatly lower in the NAVA group than the PSV group, 16 patients (26.7%) in the PSV group Vs. 6 patients (10%) in the NAVA group (*P* 0.02), (Fig. 2). NAVA group showed highly statistically significant (*P* < 0.001) lower values than that of the PSV group regarding the total asynchrony index (6.12 ± 1.51 Vs. 7.06 ± 1.25%), ineffective trigger index (0.36 ± 0.68 Vs. 3.33 ± 1.63%), auto trigger index (0.07 ± 0.18 Vs. 1.68 ± 0.83%) and prolonged cycle index (0.84 ± 1.37 Vs. 1.92 ± 1.01%). The NAVA group showed highly statistically significant (*P* < 0.001) higher values than those of the PSV group regarding double trigger index (3.16 ± 1.19 vs. 0.53 ± 0.76%) and short cycle index (1.84 ± 1.23 vs. 0.30 ± 0.75%) (Fig. 3).

### Lung mechanics: respiratory variables

There was no statistically difference between both groups regarding heart rate HR, mean blood pressure *P (*0.59, 0.83*)*, and lung mechanics including peak inspiratory pressure PIP, mean airway pressure P mean, PEEP, driving pressure and compliance *P (*0.10, 0.76, 0.39, 0.21, and 0.10*)* respectively (Table 2). Regarding respiratory variables, the tidal volume was higher in the PSV group than the NAVA group (7.15 ± 0.37 Vs. 6.70 ± 0.29 ml/Kg; *P* < 0.001), The respiratory rate RR was statistically significantly higher in the NAVA group than PSV group (19.84 ± 1.69 Vs. 19.27 ± 1.13 breath/min; *P* 0.03) and the minute ventilation MV was comparable with no difference between NAVA group and PSV group (8.06 ± 0.92 Vs. 8.26 ± 0.92 l/min; *P* 0.25), (Table 2).

**Table 2:** Hemodynamics, respiratory variables and lung mechanics among the studied groups

Variable	Group 1 PSV (*n* = 60)	Group 2 NAVA(*n* = 60)	*P* value
Heart rate (b.*p*.m)	96.84 ± 7.67	98.11 ± 16.48	0.59
Mean blood pressure (mmHg)	84.77 ± 8.99	84.44 ± 7.71	0.83
Tidal Volume (ml/Kg)	431.93 ± 48.87	406.04 ± 36.63	< 0.001^*^
Respiratory Rate (breath/min)	19.27 ± 1.13	19.84 ± 1.69	0.03^*^
Minute Ventilation (l/min)	8.26 ± 0.92	8.06 ± 0.92	0.25
Peak Inspiratory Pressure PIP (cmH2O)	18.44 ± 0.92	18.05 ± 1.58	0.10
Mean Airway Pressure P mean (cmH_2_O)	11.40 ± 0.97	11.34 ± 1.40	0.76
Positive Airway Pressure PEEP (cmH2O)	6.26 ± 0.94	6.13 ± 0.76	0.39
Driving pressure (cmH_2_O)	12.21 ± 0.83	11.93 ± 1.50	0.21
Compliance (ml/ cmH2O)	44.99 ± 3.86	46.22 ± 4.22	0.10
NAVA level in NAVA group		1.86 ± 0.28	
Edi Max (µV) in NAVA group		11.02 ± 1.84	
Edi Min (µV) in NAVA group		3.03 ± 2.30	
Pressure Support level in PSV group	10.61 ± 0.75		
The number of cases with auto PEEP	9 (15%)	3 (5%)	0.06

Data were taken every hours and this average. Values are mean ± SD.NAVA: Neurally Adjusted Ventilatory Assisted, PSV: Pressure Support Ventilation. Edi: diaphragm’s electrical activity

Data were normally distributed; tested by T-test

* = Significant difference between NAVA group and PSV group

### Oxygenation and correlations with respiratory variables

NAVA group had a highly statistically significant higher PaO_2_ (208.53 ± 14.61 vs. 188.8 ± 25.55 mmHg; *P* < 0.001) and PaO_2_/FiO_2_ ratio (521.32 ± 36.52 vs. 472.01 ± 63.88; *P* < 0.001) than the PSV group (Table 3). In PSV group, the pressure support level had a highly statistically significant positive correlation with the tidal volume (r 0.44; *P* < 0.001) and with minute ventilation (r 0.44; *P* < 0.001), with no statistically significant correlation with respiratory rate (r 0.002; *P* 0.99), ineffective trigger index (r -0.005; *P* 0.97), auto trigger index (r -0.08; *P* 0.55), double trigger index (r 0.07; *P* 0.61), prolonged cycle index(r 0.12; *P* 0.35), short cycle index (r 0.07; *P* 0.59) and total ASI (r 0.14; *P* 0.27). In NAVA group, the NAVA level had a statistically significant positive correlation with the ineffective trigger index (r 0.30, *P* 0.02) and a highly statistically significant negative correlation with delta Edi (r -0.53, *p* < 0.001) and had no statistically significant correlation with the Edi max (r 0.10; *P* 0.43), Edi min (r 0.07;P 0.58), the tidal volume(r -0.009; P 0.94), respiratory rate (r -0.10; *P* 0.44), minute ventilation (r -0.08;*P* 0.55), auto trigger index (r 0.22; *P* 0.10), double trigger index (r 0.10; *P* 0.44), prolonged cycle index (r 0.04; *P* 0.77), short cycle index (r -0.12; *P* 0.36) and total ASI (r 0.11; *P* 0.40).

**Table 3:** Arterial blood gases parameters among the studied cases

Variable	Group 1 PSV (*n* = 60)	Group 2 NAVA (*n* = 60)	*P* value
pH	7.36 ± 0.02	7.38 ± 0.02	< 0.001^*^
PaCO_2_ (mmHg)	36.27 ± 1.42	36.91 ± 2.33	0.13
PaO_2_ (mmHg)	188.8 ± 25.55	208.53 ± 14.61	< 0.001^*^
HCO_3_ (mEq/L)	21.51 ± 0.72	22.97 ± 0.76	< 0.001^*^
SpO_2_%	98.76 ± 0.42	99.01 ± 0.30	< 0.001^*^
PaO_2_/FiO_2_ Ratio	472.01 ± 63.88	521.32 ± 36.52	< 0.001^*^

NAVA: Neurally Adjusted Ventilatory Assisted, PSV: Pressure Support Ventilation

Data were taken every hours and this average. Data were normally distributed; tested by T-test

* = Significant difference between NAVA group and PSV group

### Auto PEEP frequencies among the studied groups

The number of cases with auto-PEEP (Auto PEEP frequency) was less in the NAVA group than in the PSV group, with 9 (15%) cases Vs. 3 (5%) cases but not statistically significant (*P* 0.06). (Table 2).

In the PSV group, the total asynchrony index and the ineffective trigger index were statistically significantly higher (8.12 ± 1.29 Vs. 6.87 ± 1.16%; *P* < 0.008) and (4.75 ± 1.21 Vs. 2.43 ± 0.97%; *P* < 0.001) in cases with auto-PEEP than in cases without auto-PEEP.

In the NAVA group, there was no statistically significant difference in the total asynchrony index (6.0 ± 0.67 vs. 6.13 ± 1.54%; *P* = 0.80) and the ineffective trigger index (0.44 ± 0.38 vs. 0.29 ± 0.58%; *P* = 0.34) between cases with auto-PEEP and cases without auto-PEEP.

Application of post-extubation CPAP was statistically significantly higher in cases with auto-PEEP than in cases without auto-PEEP in the PSV group (6 Vs 3 cases; *P* 0.04) and the NAVA group (1 vs. 0 cases; *P* < 0.001).

### Duration of mechanical ventilation, sedating drugs, and ICU stay

NAVA group had a statistically significantly lower duration of mechanical ventilation than the PSV group (4.05 ± 0.29 vs. 4.62 ± 0.52 h; *P* < 0.001), respectively. The range of mechanical ventilation duration hours was 4–6 in the PSV group vs. 3–5 h. in the NAVA group, including the period of spontaneous breathing trial performed before extubation. Total doses of fentanyl (123.33 ± 28.32 vs. 153.33 ± 38.91 µg) and propofol (175.0 ± 24.32 vs. 201.83 ± 32.07 mg) were used during the period of weaning in the NAVA and PSV groups, respectively.

There was no significant difference regarding the total dose of norepinephrine used, ICU stay, hospital stay in days, incidence of chest infection, and lung collapse between the two groups (*p* 0.18, 0.15, 0.08, and 0.15), respectively. (Table 4)

**Table 4:** Outcomes in both studied groups

Variable	Group 1 PSV (*n* = 60)	Group 2 NAVA(*n* = 60)	Test	*P* value
Duration of mechanical ventilation (hours)	4.62 ± 0.52	4.05 ± 0.29	t-test 7.35	< 0.001^*^
Post extubation CPAP Yes /No	6/54	2/58	Z 2.14	0.27
Duration of CPAP (hours)	*N* = 6 24.0 ± 0.0	*N* = 2 36.0 ± 16.97	U 1.73	0.08
Use of norepinephrine Yes /No	14/46	9/51	χ2 1.34	0.25
Total Dose of norepinephrine (mg)	*N* = 14 1.31 ± 0.46	*N* = 9 1.11 ± 0.32	U 1.34	0.18
Total dose fentanyl (µg)	153.33 ± 38.91	123.33 ± 28.32	t-test 4.83	< 0.001^*^
Total dose propofol (mg)	201.83 ± 32.07	175.0 ± 24.32	t-test 5.16	< 0.001^*^
ICU Stay (days)	2.80 ± 0.40	2.68 ± 0.47	t-test 1.46	0.15
Hospital Stay (days)	8.5 ± 0.89	8.25 ± 0.60	t-test 1.80	0.08
Chest infection Collapse Pancreatic leak	9 (15.0) 8 (13.3) 1 (1.7)	8 (13.3) 2 (3.3) 0 (0.0)	Z 5.35	0.15

NAVA: Neurally Adjusted Ventilatory Assisted, PSV: Pressure Support Ventilation

Data normally distributed; tested by T-test. Data not normally distributed tested by Mann-Whitney U test. Chi-squared test (χ2), Fisher’s exact (Z)

* = Significant difference between NAVA group and PSV group

### ROC curve analysis for asynchrony index prediction of the need for postoperative CPAP

**Fig. 4:**
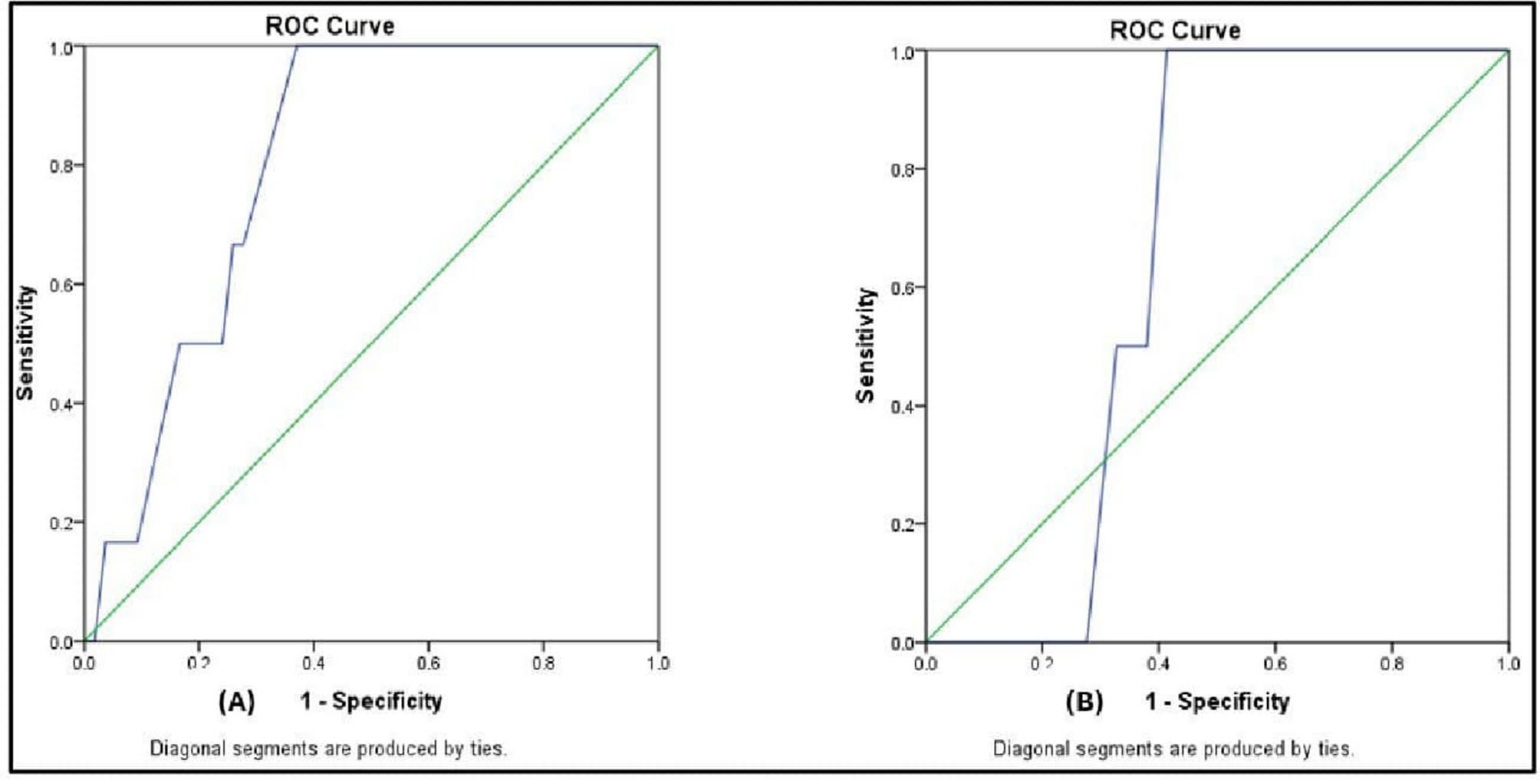
The ROC curve for the total asynchrony index ASI for prediction of the need for postoperative CPAP in PSV group (**A**) and in NAVA group (**B**)

NAVA reduced the need for post-extubation CPAP, but the difference was not statistically significant between the groups. ROC curve analysis for asynchrony index for prediction of the need for postoperative CPAP in PSV group demonstrated AUC 0.80 with *P* 0.016 (95% CI: 0.68–0.93), at a cutoff point of 7.125, the sensitivity was 100%, specificity was 63%, positive predictive value 23.1% and negative predictive value 100%, (Fig. 4A). While in NAVA group, its AUC 0.65 with *P* 0.47 (95% CI: 0.51–0.79), at a cutoff point 5.83, its sensitivity was 100%, specificity was 58.6%, positive predictive value 8.3% and negative predictive value 100%, (Fig. 4B).

## Discussion

In our study, the fundamental outcome is that the total asynchrony index ASI and the number of cases with extreme asynchrony ASI ≥ 10% were statistically significantly lower in the NAVA group than in the PSV group, demonstrating that NAVA provides better patient-ventilator interaction and synchronization than PSV. This because PSV assisting each breath with a constant pre-set level of pressure regardless of the inspiratory effort of the patient [3], However NAVA tailors the assistance level conveyed by the ventilator according to the electrical activity of the diaphragm Edi which is a guidance of the inspiratory effort of the patient in a dynamic synchronized manner for each breath rather than a pre-set fixed manner for all breaths [15]. Kyo M, et al., in a systematic review and meta-analysis, defined severe asynchrony as total ASI ≥ 10% 10%. As this value causes patients’ discomfort, augments the work of breathing, and delays weaning, it leads to exhausted diaphragmatic energy, increases the duration of mechanical ventilation, and increases the mortality rate.^[ 5]^ So, we defined severe asynchrony as total ASI ≥ 10% based on the previous studies and its ability to differentiate clinically related asynchrony and its relation to adverse clinical consequences.

This result corresponds to Lamouret et al.‘s outcome [16], who stated that the total asynchrony index was reduced in NAVA than in PSV mode. Spinazzola et al. [17] carried out a crossover study in pediatric patients with moderate acute respiratory distress syndrome ARDS and recorded three study periods: PSV1, NAVA, and PSV2, each lasting for 1 h. They also found that the asynchrony index was significantly lower in the NAVA period than in the PSV1 and PSV2 periods. Sehgal et al. [18] concluded that the mean difference between the asynchrony index and the occurrence of severe asynchrony was significantly higher during PSV mode in both adult and pediatric populations during non-invasive ventilation for acute respiratory failure. On the contrary, Diniz-Silva et al. [14] conducted a prospective randomized study on patients with ARDS shifting from controlled ventilation to partial ventilatory support. They applied either NAVA or PSV for 15 min, followed by NAVA for 3 h, and found no difference between NAVA and PSV in the asynchrony index. The contrast between their results and ours is most probably due to different numbers and types of patients we studied, 120 post-surgical patients with healthy lungs as they studied only 15 patients suffering from ARDS that were ventilated with low tidal volumes, which limited the development of ineffective triggering which is the most prevalent type of asynchrony.

In our study, the ineffective trigger index was statistically significantly lower in the NAVA group than in the PSV group. As the breath in NAVA is triggered by the EDI signal, not by initiating negative pressure or flow as in PSV, there is less possibility for missed effort, and it can occur only if the captured EDI signal is very low due to respiratory center inhibition, as in heavy sedation or hypocarbia [19]. In our study, trigger sensitivity was set at (− 1 Cm H_2_O) (the most sensitive trigger to initiate breath), so no intervention or further adjustments were done when ineffective triggers were detected, just recording the frequency and comparing them between both groups. Spinazzola et al. [17], Diniz-Silva et al. [14], and Demoule et al. [20] concluded the same as our results that NAVA reduced the ineffective triggering. Lamouret et al. [16], who reported no difference between NAVA mode and PSV mode in ineffective triggering. Their results are against ours as they studied both modes on the same patient, each mode for one hour, and analyzed only eight periods, 1 min each for both modes.

Our results showed that the auto-trigger index is statistically greater in the PSV group than in the NAVA group. Auto triggering can occur in PSV due to air leaks, an extremely high trigger sensitivity, alterations in airway flow or pressure or due to cardiac oscillations, or water collection in ventilator tubing that can perceived as triggering efforts during PSV [21], but NAVA is only triggered by the Edi signal, not by changes in airway pressure or flow and auto triggering can occur only if there are artifacts on the Edi signal [22]. Our result is compatible with Spinazzola et al. [17] and Diniz-Silva et al. [14], who concluded higher auto-triggering during PSV than during NAVA. On the other hand, Lamouret et al. [16] and Ferreira J et al. [23] showed no significant differences in auto-triggering between NAVA and PSV. Their studies included smaller sample sizes and shorter recording periods than this study, yielding differences in the results.

Our results showed that the double trigger index is higher in the NAVA group than in the PSV group. Double triggering in NAVA is correlated to the EDI signal that shows a biphasic curve, and the ventilator cycles off when the EDI drops to 70% of its peak; afterward, a recovery in inspiratory flow causes the retriggering [24]. Double triggering impacts patient safety as it increases the work of breathing, patient discomfort, ineffective ventilation, and gas exchange due to air trapping. All previous effects come back to the frequency of their occurrences. Our results showed that the double trigger index is only (3.16 ± 1.19vs 0.53 ± 0.76%; *p* < 0.001) in the NAVA and PSV group, respectively, which has no significant impact on patient safety. Our results are similar to Lamouret et al. [10], Ferreira et al. [23], and Piquilloud et al. [25], who stated that double triggering was more common in NAVA than in PSV. Di Mussi et al. [26] reported no significant difference in double triggering and short cycle indecis in NAVA and PSV groups. They studied fewer patients ventilated with controlled mechanical ventilation for 72 h, while we studied post-surgical patients, yielding different results.

The cycling asynchronies depend on the cycling criteria set in each mode; it was set at 70% of the EDi max in NAVA and 25% of the peak flow in PSV [10]. Our results demonstrated that delayed cycling (prolonged cycle) was higher in the PSV group than the NAVA group because the flow may decelerate more slowly than expected and cause the breath to cycle late during PSV. Premature cycling (short cycle) was not frequent but higher in the NAVA group than the PSV group, as explained before, with double triggering, as triggering and cycling are related to the Edi signal that sometimes shows a biphasic curve [24]. Demoule et al. [20] observed a similar finding that late cycling was more frequent in the PSV group than the NAVA group. Piquilloud et al. [25] reported that no late cycling occurred with NAVA and less premature cycling with NAVA than in PSV. Lamouret et al. [16] reported less late-cycle and premature cycling in NAVA mode than in PSV mode. On the other hand, Ferreira et al. [23] stated that cycling delay and premature cycling were comparable for both PSV and NAVA.

Regarding respiratory parameters, the respiratory rate RR was statistically greater in the NAVA group than in the PSV group. There was more ineffective triggering in the PSV greater in the PSV group than in the NAVA group. As in NAVA, the level of assistance delivered was correlated to the EDI, which is a surrogate of the patient’s respiratory center, while in PSV, the pressure is preset and fixed. So, NAVA avoids over-assistance and higher TV in contrast to PSV. The minute ventilation MV was comparable between the two groups. As the minute ventilation is dependent on both RR and TV, and due to differences between the two groups in RR and TV, the net result is similar minute ventilation between the two groups. Piquilloud et al. [25] reported a similar finding that tidal volume was less in NAVA than in PS1 and PS2, and respiratory rate was higher in NAVA than in PS1 and PS2 without a difference in minute ventilation between NAVA and PS. Also similar to Lamouret et al. [16], found that the tidal volume was lower in NAVA than in PSV and the respiratory rate was greater in NAVA than in PSV. Demoule et al. [20], Ferreira et al. [23], and Diniz-Silva et al. [14], observed that tidal volume, respiratory rate, and minute ventilation did not differ between the two groups. The peak inspiratory pressure PIP was statistically greater in the PSV group than the NAVA group because PIP in NAVA differs with Edi; compared to PSV, it is pre-defined. Our results found that mean airway pressure and compliance were similar in the two groups. This is consistent with Piquilloud et al. [25] who found that the mean airway pressure was reduced with NAVA. In contrast, Diniz-Silva et al. [14] stated that the PIP was larger in NAVA than in PSV but stayed within safe levels in both modes.

Our result found that the NAVA group had statistically significantly higher PaO2 (208.53 ± 14.61 vs. 188.8 ± 25.55 mmHg; *p* < 0.001) and PaO2/FiO2 ratio (521.32 ± 36.52 vs. 472.01 ± 63.88; *p* < 0.001) than the PSV group. Despite these significant differences, both modes, PSV and NAVA, achieved good oxygenation for the patients, and can’t translate that difference to be the reason for the different patient outcomes. This point needs further assessment in future studies.

Our results demonstrated that the total doses of fentanyl and propofol used during the period of weaning were lower in the NAVA group than in the PSV group. Because NAVA is more synchronous with the patient’s respiratory effort and demand through the EDI, it decreases the fighting against the ventilator and sedation requirements. Similarly, Sood et al. [27] conducted a study of pediatric patients undergoing cardio-pulmonary bypass surgery that requires more than 96 h of postoperative ventilation. They used NAVA, or SIMV-PRVC + PSV, to wean these patients. They reported a significant reduction in sedative use in the NAVA. On the other hand, Hadfield et al. [28] carried out a study comparing NAVA and PSV for weaning in patients with prolonged mechanical ventilation; they found that total sedation used was similar in the NAVA group and the PSV group.

Our results showed that the duration of mechanical ventilation in hours was highly statistically significantly lower in the NAVA group than the PSV group with no difference regarding ICU stay and hospital stay in days and complications such as chest infection or lung collapse between the two groups Similarly, Chen et al. [29], concluded that NAVA significantly reduced the duration of ventilation than the PSV, but there were no significant differences in ICU stay time and hospital stay time. In contrast, Weiyun et al. [30] reported longer ICU stays in NAVA than PSV.

Our results showed that the number of cases with auto-PEEP occurrences was reduced in the NAVA group. There were 9 (15%) in the PSV group and 3 (5%) cases in the NAVA group. So, we concluded that NAVA lowers the risk of auto-PEEP occurrence. Because the tidal volume and the inspiratory and expiratory times of the ventilator are personalized by the patient’s respiratory canter via the EDI signal, optimizing ventilation and decreasing the risk of air trapping. Our results demonstrated that in the PSV group, the total asynchrony index and the ineffective trigger index were significantly higher in cases with auto-PEEP than in cases without it. Patients must overcome the auto-PEEP due to air trapping to reach the trigger level in PSV, resulting in increased ineffective efforts and total asynchrony index. The application of post-extubation CPAP was significantly higher in cases with auto-PEEP than in cases without. Our results showed that in the NAVA group, there was indistinguishability in the total asynchrony index and the ineffective trigger index between cases with auto-PEEP and cases without auto-PEEP. Because in NAVA, the ventilator is triggered by the EDI signal independent of air trapping, NAVA improves trigger asynchronies in patients with auto-PEEP. The application of post-extubation CPAP was significantly higher in cases with auto-PEEP than in cases without auto-PEEP.

Similarly, Giacomo Bellani et al. [31] carried out a study on 10 intubated patients ventilated with PSV with a suspicion of intrinsic PEEP to assess whether NAVA was advantageous in contrast with PSV in patients with auto-PEEP. They concluded that NAVA decreased the pressure essential to overwhelming auto-PEEP and provided a better patient-ventilator synchrony than PSV in patients with relevant auto-PEEP. Weiyun et al. [30] found that the AI of NAVA was significantly lower than PSV in COPD exacerbation and non-COPD patients. Also, Piquilloud et al. [25] found that NAVA improved asynchrony in chronic obstructive pulmonary disease patients, but auto-PEEP wasn’t measured.

Our results showed that post-extubation CPAP was needed in 6 patients (10%) in the PSV group and only two (3.3%) in the NAVA group. So, NAVA reduced the need for post-extubation CPAP, but this was not statistically significant. Demoule et al. [20] observed a similar finding that NAVA significantly decreased the application of post-extubation non-invasive mechanical ventilation. Our study has some limitations: First, patients did not need ventilation for a longer duration, which would have given us enough time to prove more benefits for NAVA. We recommend applying NAVA ventilation for patients requiring ventilation for longer durations in further studies. Second, the software for NAVA ventilation is only available for the Maquet Servo-I ventilator, which hinders the application of NAVA ventilation in the intraoperative period. Third, we did not have automatic software for detecting synchronous breath in an easy and precise way. Fourth, the Edi catheter is only for single use and cannot be sterilized and reused, increasing the cost. Finally, as after approval of the local Menoufia University Faculty of Medicine research ethics committee IRB (N. and date 3/2020/ANET9), there were many challenges in providing NAVA cables and module so unintentionally we forgot to make clinical trial registration we thought we make it, when we start to write and proceed in the publication we discovered we did not make it so we make the retrospective registration at the Pan African Clinical Trial in 26 January 2024.

## Conclusions

In hepatic patients undergoing Whipple operation, NAVA serves better patient-ventilator interaction and synchronization than PSV, as indicated by a lower total asynchrony index, less number of patients with total ASI ≥ 10%, lowered risk of auto-PEEP occurrence, minimized need for post-extubation CPAP, reduced total doses of fentanyl and propofol used during the period of weaning, and decreased the duration of mechanical ventilation needed.

## Electronic supplementary material

Below is the link to the electronic supplementary material.

Supplementary Material 1 Supplementary Material 1

Supplementary Material 2 Supplementary Material 2

Supplementary Material 3

## Data Availability

The datasets used and/or analyzed during the current study available from the corresponding author on reasonable request.

## Abbreviations

ICU: Intensive care unit
NAVA: Neurally Adjusted Ventilatory Assisted
PSV: Pressure Support Ventilation
Edi: Diaphragm’s electrical activity
ASI: Asynchrony index
RASS: Richmond Agitation & Sedation Scale
