# Supplementary figures and images for: Neurally adjusted ventilatory assisted ventilation compared to pressure support during post-operative weaning of hepatic patients undergoing major abdominal surgeries: a randomized control trial
Source: BMC Anesthesiol. 2025 Jun 26;25:297. doi: 10.1186/s12871-025-03159-y (PMC12199501; doi:10.1186/s12871-025-03159-y)

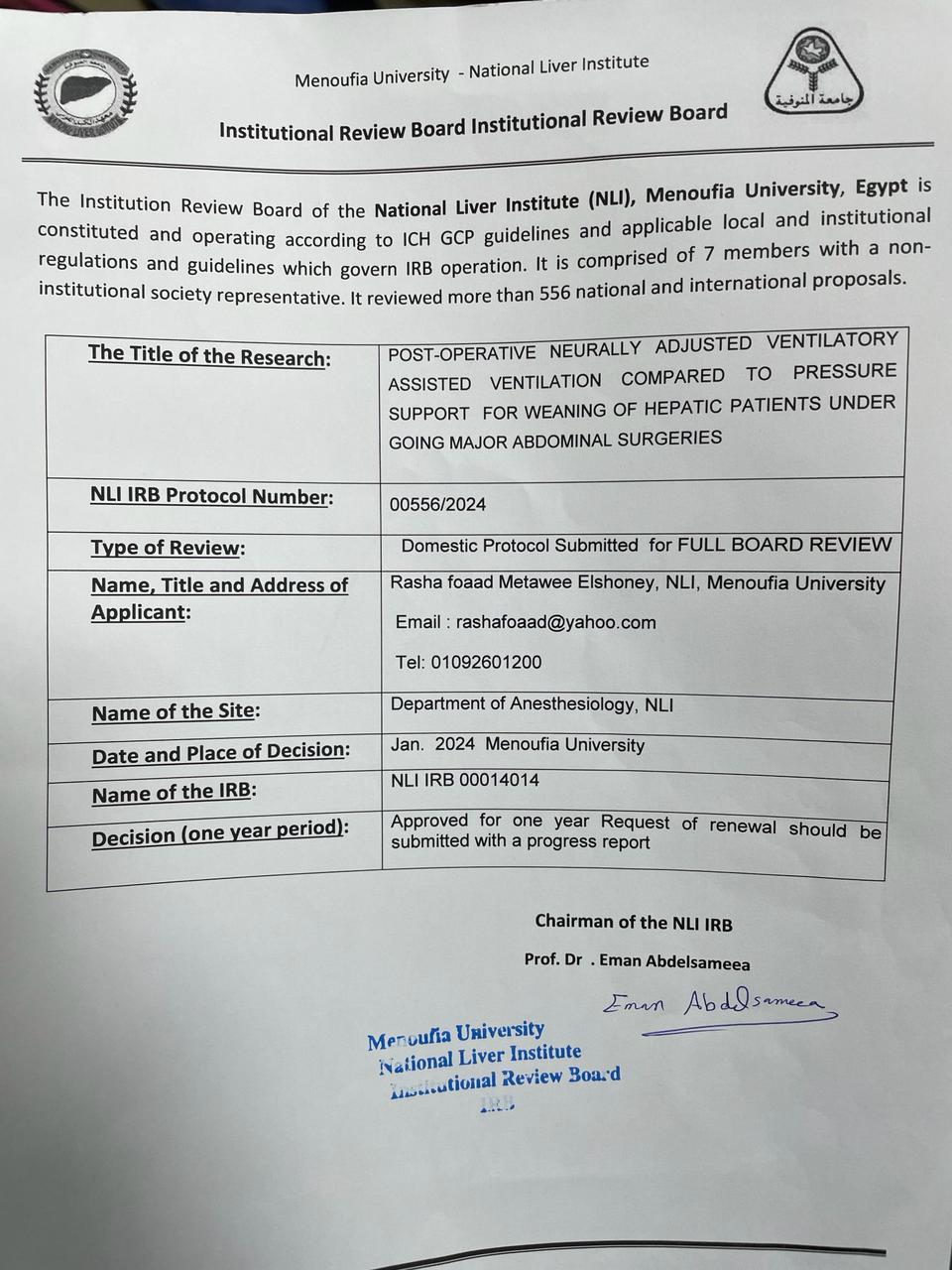

Supplement: Supplementary file 1 — Supplementary Material 1 [file 12871_2025_3159_MOESM1_ESM.jpg]

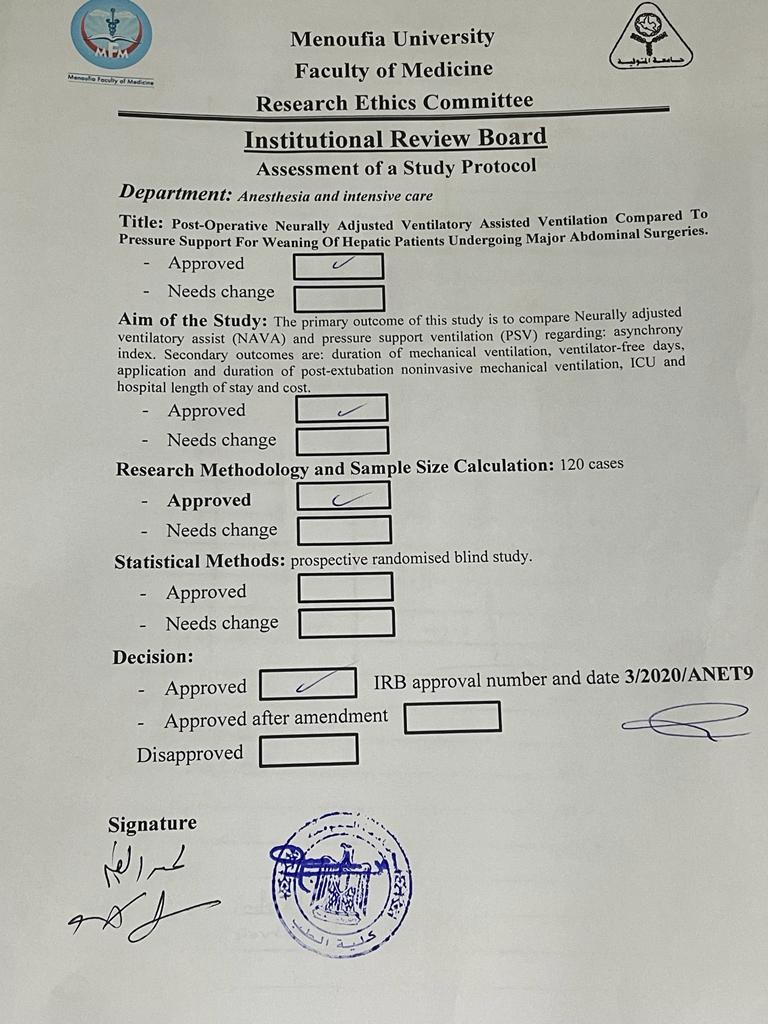

Supplement: Supplementary file 2 — Supplementary Material 2 [file 12871_2025_3159_MOESM2_ESM.jpg]
